# Comparison of the trapezius and the adductor pollicis muscle as predictor of good intubating conditions: a randomized controlled trial

**DOI:** 10.1186/s12871-017-0401-8

**Published:** 2017-08-17

**Authors:** Stefan Soltesz, Christian Stark, Karl G. Noé, Michael Anapolski, Thomas Mencke

**Affiliations:** 1 Department of Anesthesia and Intensive Care Medicine, KKH Dormagen, Dormagen, Germany; 2 Department Ob/Gyn, University of Witten-Herdecke, KKH Dormagen, Dormagen, Germany; 3 Department of Anesthesia and Intensive Care Medicine, University of Rostock, Rostock, Germany; 4 Klinik für Anästhesie, Intensiv- und Notfallmedizin, Kreiskrankenhaus Dormagen, D-41540 Dormagen, Germany

**Keywords:** Neuromuscular block, Intubating conditions, Trapezius muscle

## Abstract

**Background:**

Adequate muscle relaxation is important for ensuring optimal conditions for intubation. Although acceleromyography of the adductor pollicis muscle is commonly used to assess conditions for intubation, we hypothesized that acceleromyography of the trapezius is more indicative of optimal intubating conditions. The primary outcome was the difference between both measurement sites with regard to prediction of good or acceptable intubating conditions.

**Methods:**

Neuromuscular blockade after injection of rocuronium 0.3 mg/kg IV was measured simultaneously with acceleromyography of the adductor pollicis muscle and the trapezius muscle in sixty female patients, American Society of Anesthesiologists physical status I to III, undergoing general anesthesia for gynecologic surgery. Exclusion criteria were: expected difficult tracheal intubation (e.g. history of difficult intubation, reduced mouth opening (< 2 cm) and/or Mallampati Score 4), increased risk of pulmonary aspiration (e.g. gastroesophageal reflux or delayed gastric emptying) allergies to drugs used during the study, pregnancy, neuromuscular diseases, medication with potential to influence neuromuscular function (e.g. furosemide, magnesium, cephalosporins) and hepatic or renal insufficiency (serum bilirubin >26 μmol/L, serum creatinine >90 μmol/l). Patients were randomized to 2 groups: group A (*n* = 30): endotracheal intubation after onset of the neuromuscular block at the adductor pollicis muscle. Group B (*n* = 30): endotracheal intubation after onset at the trapezius muscle. Intubating conditions were compared between both groups by means of a standardised score (the Copenhagen score) with Fisher’s exact test.

**Results:**

Onset of the block after rocuronium injection was observed at the adductor pollicis muscle compared to the trapezius with 2.8 (1.1) versus 2.5 (1.1) min (mean ± SD; *P* = 0.006). Intubating conditions were poor in 2 patients (7%) of group A, and in 1 patient (3%) of group T. They were acceptable (either excellent or good) in 28 patients (93%) in group A, and in 1 patient (97%) in group T (*P* = 0.82).

**Conclusions:**

Performing acceleromyography at the trapezius muscle reduced the time between injection of neuromuscular blocking agents and intubation by 18 s (11%). Thus, trapezius muscle acceleromyography is an acceptable alternative to adductor pollicis muscle acceleromyography in predicting acceptable intubating conditions, which allows for earlier indication of adequate intubating conditions.

**Trial registration:**

ClinicalTrial.gov Identifier: NCT01849198. Registered April 29, 2013.

## Background

An adequate neuromuscular block improves intubating conditions and increases the rate of successful intubation. Thus, the risks of laryngeal injuries or pulmonary aspiration are reduced [1]. Unfortunately, neuromuscular measurements at the adductor pollicis muscle, probably the location used most frequently in the clinical routine, correlate poorly with intubating conditions. The reason for this observation might be that onset is slower and resistance to neuromuscular blocking agents is less compared to diaphragm or larynx [2–4].

Acceleromyography of the adductor pollicis muscle is commonly used to assess for adequate neuromuscular blockade. However, the onset of a neuromuscular block varies considerably depending on the muscle group used for assessment**:** for example**,** compared to the adductor pollicis muscle, a faster onset could be observed at the larynx [4], diaphragm, or masseter muscle [5, 6]. Correlation between acceleromyography and intubating conditions were better with the masseter than with the other muscles. Therefore, the ability to predict maximum neuromuscular block with acceleromyography depends on the muscle site used for acceleromyographic assessment.

In this study, a recently introduced location for acceleromyography – the trapezius muscle - was examined and compared to a simultaneous stimulation of the adductor pollicis muscle [7]. Onset and recovery at the trapezius muscle were faster than at the adductor pollicis muscle. Therefore, the present study was performed to compare these two muscles with regard to prediction of good intubating conditions. Our hypothesis was that the trapezius muscle might reflect the laryngeal muscles better because of its proximal localization and might therefore be an alternative to the adductor pollicis muscle. The primary outcome was the percentage of good or excellent intubating conditions at both measurement sites.

## Methods

### Patient selection

After approval of the local ethics committee and having obtained written informed consent (Ethikkommission der Ärztekammer Nordrhein, Duesseldorf, Germany, April 23th, 2013; No 2013056), we performed this prospective, unblinded, single center, randomized and controlled study (ClinicalTrial.gov Identifier: NCT01849198). We recruited female patients from 18 to 65 years, American Society of Anesthesiologists physical status I-III, body weight 50 to 90 kg, undergoing elective laparoscopic gynecological surgery.

Exclusion criteria were: expected difficult tracheal intubation (e.g. history of difficult intubation, reduced opening of the mouth (< 2 cm) and/or Mallampati Score 4), increased risk of pulmonary aspiration (e.g. gastroesophageal reflux or delayed gastric emptying) known allergies to drugs administered during the study, pregnancy, neuromuscular diseases, medication with potential to influence the neuromuscular function (e.g., furosemide, magnesium or cephalosporins) and hepatic or renal insufficiency (serum bilirubin >26 μmol/L, serum creatinine >90 μmol/l).

### Anesthesia

All patients were pre-medicated with midazolam 7.5 mg per os. In the operating room, they received 100% oxygen via facemask together with an intravenous infusion of remifentanil 0.2 μg kg^−1^ min^−1^. Induction of anesthesia was performed with fentanyl 2 μg/kg and propofol 2–3 mg/kg. Initially a laryngeal mask (Ambu® AuraOnce™ size 4, Ambu Inc., Glen Burnie, MD 21060 USA) was inserted and anesthesia was maintained by continuous infusion of remifentanil 0.15–0.25 μg/kg/min and propofol 3–5 mg/kg/h. Eventually the laryngeal mask was replaced by an endotracheal tube (Rueschelit® 7.0 mm I.D., TeleflexMedical, Athlone, Ireland) after onset of the neuromuscular block**.** Patients’ lungs were ventilated with a respiratory rate of 10–12/min and a tidal volume of 6–8 ml/kg in order to achieve normocapnia, defined as an end-tidal CO_2_ concentration of 36–40 mmHg. Hemodynamic parameters were maintained within ±20% of baseline values.

**Fig. 1:**
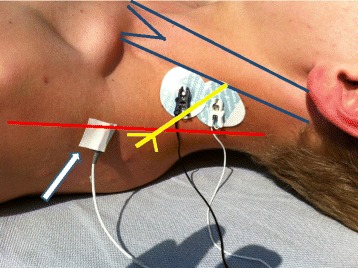
Position of the electrodes at the accessory nerve and the piezoelectric element (white arrow) on the trapezius muscle. Blue lines: sternocleidomastoid muscle. Red line: anterior border of the trapezius muscle. Yellow lines: accessory nerve

After placement of the laryngeal mask, neuromuscular transmission was measured by simultaneous monitoring of acceleromyographic responses (TOF Watch SX, Essex Pharma GmbH, Munich, Germany) at the adductor pollicis muscle and the trapezius muscle. Both muscles were stimulated using transcutaneous Ag/AgCl electrodes (electrocardiogram electrodes; Ambu Inc., MD 21060 USA) placed at the wrist of the right hand, and 1 cm dorsal to the inferior border of the left sternocleidomastoid muscle, respectively (Fig. 1). The right hand was fixed while the thumb was free to move [8]. Thus, contractions of the hand influencing the measurements at the thumb during ulnar stimulation were avoided. With regard to the accessory nerve, adequate stimulation was verified by measuring the movements of the left shoulder in a cranial direction.

The accelerometers’ piezoelectric elements were fixed distally at the right thumb [8], and at the left trapezius muscle in a distance of approximately 10 cm from the electrodes (Fig. 1). Both accelerometer were calibrated to deliver a supramaximal train of four (TOF) stimulus (0.2 Hz every 15 s, duration 0.1 ms). The TOF Watch SX automatically determines the individual supramaximal stimulation current (up to a maximum current of 60 mA). These maximal acceleromygraphic responses served as control values [9]. The first of the four twitch height responses was regarded to be T1, and the TOF ratio was calculated as the ratio of the fourth twitch (T4) height response and T1. During the first minutes of the acceleromyography, the electric current of the stimulation often changes the impedance of the electrodes thereby causing a drift of the acceleromyographic responses. Therefore, both acceleromyographs were recalibrated 10 min after start of the first stimulation.

After a constant signal had been established at both measurement sites, rocuronium 0.3 mg/kg *iv* was injected over a period of 5 s. Directly afterwards, the intravenous line was flushed with Lactated Ringers’ solution, and neuromuscular block height was measured simultaneously at both locations every 15 s. The obtained data were stored on 2 computers which were connected to the TOF Watch SX devices (TOF-Watch SX Monitor Version 2.5.INT; Organon Ltd., Dublin, Ireland). Onset time and maximum block height were obtained as recommended by Fuchs-Buder et al. [8].

**Fig. 2:**
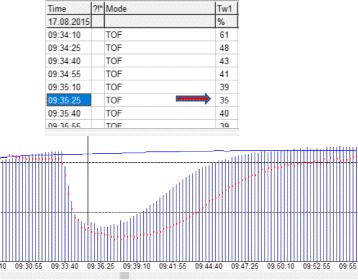
Measurement of onset time in case of an incomplete neuromuscular block (defined as T1 height did not fall <5% of baseline values): in case of an incomplete onset time was measured as the time from start of injection of rocuronium until at least 3 consecutive twitches with the same or even increasing amplitude were observed. The first of these T1 twitches served as endpoint for calculation of the onset time (red arrow and black vertical line, respectively)

Onset time was defined as follows: Time from start of injection of rocuronium until T1 height fell <5% compared to baseline values. In case of an incomplete block (minimal T1 height 5% or higher compared to baseline values), onset time was measured as the time from start of injection of rocuronium until at least 3 consecutive twitches with the same or even increasing amplitude were observed. The first of these T1 twitches served as endpoint for calculation of the onset time (Fig. 2).

Maximum block height was regarded as height of T1 at the onset of the block in percent compared to baseline values.

### Assessment of intubating conditions

Patients were randomized to two groups with different time points of endotracheal intubation according to a computerized allocation schedule: in group A (*n* = 30), intubation was performed when onset time was reached at the adductor pollicis muscle. In group T (*n* = 30), intubation was performed when onset time was reached at the trapezius muscle. Patients’ tracheas were always intubated by the same experienced anesthetist.

Intubating conditions were assessed by means of the following variables [8]: ease of laryngoscopy (laryngoscopy component), position and movement of the vocal cords (vocal cord component), coughing or movement of the limbs during or directly after intubation (reaction to intubation component). Each of these variables could be rated as excellent, good or poor (Table 1). Intubating conditions were rated as excellent if all variables were excellent, they were good if all variables were good or excellent, and they were poor if any variable was poor.

**Table 1:** Criteria used to assess intubating conditions. Overall intubating conditions were rated as excellent if all variables were excellent, they were good if all variables were good or excellent, and they were poor if any variable was poor [8]

Variable assessed	Clinically acceptable		Clinically not acceptable
	Excellent	Good	Poor
Laryngoscopy	Easy	Fair	Difficult
vocal cords position	Abducted	Intermediate/moving	Closed
Reaction to insertion of the tracheal tube and cuff inflation (diaphragmatic movements/coughing)	none	slight	Vigorous/sustained

Laryngoscopy: easy: jaw relaxed, no resistance to blade insertion. Fair: jaw not fully relaxed, slight resistance to blade insertion. Difficult: poor jaw relaxation, active resistance of the patient to laryngoscopy. Reaction to insertion of the tube: slight: one or two movements for less than 5 s. Vigorous/sustained: more than 2 contractions/movements for longer than 5 s

### Statistical analysis

Statistical analysis was performed with Sigma Plot 12.3 for Windows software package (Systat Software Inc., Chicago, IL).

Maximum block height and onset time of neuromuscular block were analysed with a paired t-test, because both muscles could be monitored in the same patient.

Comparison between the groups were performed with an unpaired t-test (demographic data).Non-parametric data were analysed with Fisher’s exact test (intubating conditions).

The primary outcome parameter was assessed with a subjective ranking scale. Therefore, a 33% difference between groups with regard to excellent or good intubating conditions was regarded as clinically relevant. To detect this difference with *P* < 0.05 and a power of 80%, 29 patients per group were required.

## Results

**Fig. 3:**
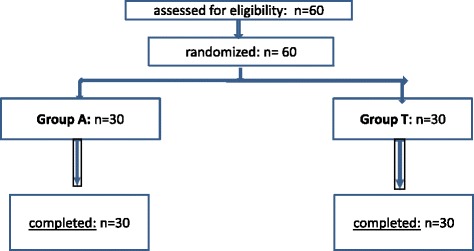
Flow chart of patient selection. Group A: measurement of intubating conditions when onset time was reached at the aductor pollicis muscle. Group T: measurement when onset was reached at the trapezius muscle

Sixty patients were included into the study between June 2013 and May 2016 (Fig. 3). The demographic data did not differ between groups and are displayed in Table 2.

**Table 2:** Data of the patients

	Group A (*n* = 30)	Group T(*n* = 30)	*P* value
age (years)	41 (8.1)	41 (6.8)	0.918
weight (kg)	67.4 (8.7)	68.7 (10.9)	0.624
height (cm)	166 (6.4)	167 (4.7)	0.715
BMI (kg/m^2^)	24.5 (3.0)	24.7 (3.9)	0.79
ASA	1.5 (0.5)	1.4 (0.5)	0.38

Data are presented as mean (SD). Group A: measurement of intubating conditions after onset of the neuromuscular block at the adductor pollicis muscle. Group T: measurement after onset at the trapezius muscle. ASA: American society of Anesthesiologists physical status

### Onset time and maximal neuromuscular block

The supramaximal stimulation currents required for the measurements were similar in both groups with 49.2 ± 11.6 mA in group A, and 49.8 ± 11.6 mA in group T, respectively (mean ± SD).

Following injection of rocuronium, maximum block was recorded slightly later at the adductor pollicis muscle compared to the trapezius muscle: 2.8 ± 1.1 versus 2.5 ± 1.1 min, respectively, (mean ± SD; *P* = 0.006)**.** The corresponding T1 twitch heights recorded did not differ: 2.8 ± 5.2% at the adductor pollicis muscle versus 1.9 ± 5.2% at the trapezius muscle (mean ± SD). Data are displayed in Table 3.

**Table 3:** Course of the neuromuscular block

	adductor muscle (*n* = 60)	trapezius muscle (*n* = 60)	*P* value
supramaximal stimulation (mA)	49,2 (11.6)	49.8 (11.6)	0.77
onset time (min)	2.8 (1.1)	2.5 (1.1)*	0.006
twitch height (% T_1_ height)	2.8 (5.2)	1.9 (5.2)	0.33

Data are presented as mean (SD). Adductor muscle: neuromuscular measurements obtained at the adductor pollicis muscle; trapezius muscle: measurements obtained at the trapezius muscle. Supramaximal stimulation: strength of supramaximal stimulation in mA; onset time: time between the beginning of injection of rocuronium and maximum T_1_ depression; twitch height: twitch height in percent compared to baseline values; n: number of successful measurements. *: *P* < 0.05 for trapezius vs. adductor pollicis

### Intubating conditions

Tracheal intubation was performed without complications or difficulties in all patients of both groups. Overall intubating conditions were poor in 2 patients (7%) of group A, and in 1 patient (3%) of group T. They were acceptable (excellent or good) in 28 (93%) of the patients in group A, and in 29 (97%) of the patients in group T (*P* = 0.82). Thus, differences with regard to intubating conditions were not observed between groups. No differences were found between groups with regard to the 3 assessed variables (laryngoscopy, vocal cords and diaphragm). Details are provided in Table 4.

**Table 4:** Intubating conditions when onset time of the neuromuscular block was reached at the adductor pollicis muscle or the trapezius muscle, respectively [8]. No significant differences between groups

Intubating conditions	Group A (*n* = 30)	Group T (*n* = 30)	*P* value
Laryngoscopy:			0.07
easy	25	30	
fair	4	0	
difficult	1	0	
Vocal cords:			0.73
abducted	26	24	
intermediate	4	6	
closed	0	0	
Diaphragmatic movements:			1.0
none	26	26	
slight	3	3	
sustained	1	1	
Overall intubating conditions			0.82
excellent	22	22	
good	6	7	
poor	2	1	

Group A: measurement of intubating conditions after onset of the neuromuscular block at the adductor pollicis muscle. Group T: measurement after onset at the trapezius muscle

## Discussion

The present study compared the intubating conditions at two different acceleromyographic measurement sites (adductor pollicis and trapezius muscles) after rocuronium administration. Intubating conditions were assessed by the Copenhagen Scoring System. This score standardises the observations in three categories thereby facilitating the data comparison from different studies. Therefore, its use is recommended for studies assessing intubating conditions [8]. Although the onset times differed from each other at the two measurement sites, intubating conditions were similar in both groups.

Several studies have been performed to find a more suitable stimulation site in order to predict optimal intubating conditions. Lee et al. performed a study assessing intubating conditions at maximal neuromuscular block at 3 different sites: the adductor pollicis muscle, the orbicularis oculi muscle and the corrugator supercilii muscle [10]**.** Twitch observation at the orbicularis oculi muscle allowed a faster intubation. Unfortunately, the frequency of inadequate conditions was increased. The authors observed best intubating conditions if the adductor pollicis muscle was chosen to predict onset of the neuromuscular block; however, with the longest delay between start of anesthesia and tracheal intubation. In a second study, they compared orbicularis oculi, corrugator supercilii, masseter and mylohyoid muscles with each other [11]. Again, best intubating conditions were observed at the measurement sites with the longest delay between induction and complete neuromuscular block (mylohyoid muscle). The authors concluded that monitoring of the corrugator supercilii muscle provided the best compromise between acceptable intubating conditions and short onset. This statement is supported by the findings of Plaud et al. observing that the corrugator supercilii muscle reflected onset of the block at the laryngeal muscles better than the orbicularis oculi muscle [12]**.**

However, the movements of the orbicularis oculi and the corrugator supercilii muscles were small; therefore, quantifying the measurements was difficult [12]**.** Finally, these muscles lie close to the corresponding facial nerves. Therefore, the risk of accidental direct stimulation of the muscle instead of the nerve is relatively high. Conversely, other authors found that monitoring onset of the neuromuscular block at the orbicularis oculi muscle would be able to predict good intubating conditions [13]. In the light of these observations, acceleromyography at the trapezius muscle might have several advantages: it is as easy to perform as e.g. measurement at the adductor pollicis muscle, it produces reliable data, and there is no need of specialized equipment [7]. Moreover, manipulations such as mask ventilation do not interfere with assessment to the same degree as with measurements at the orbicularis oculi muscle and the corrugator supercilii muscles.

Kitajima et al. compared tactile and acceleromyographic assessment of the masseter muscle with acceleromyography of the adductor pollicis muscle [5, 6]. They observed a faster onset at the masseter muscle with intubating conditions graded as good or excellent. The masseter muscle is localised near to the trapezius muscle. Therefore, these observations are consistent with the results of our investigation demonstrating similar results with a faster onset at the trapezius muscle without observing deteriorated intubating conditions.

Taken together, the literature is equivocal with regard to the optimal measurement site for assessment of good intubating conditions. Thus, further research addressing this topic would be of interest.

Studies assessing intubating conditions are methodologically difficult to perform, because it is difficult to quantify the evaluation. In our study we adhered to the recommendations for studies assessing neuromuscular blocking agents [8]. We evaluated intubating conditions by the Copenhagen score introduced by Viby-Mogensen [14] in order to make our results comparable to the data of other authors. In addition, we used a relatively low dose of rocuronium making it easier to detect small differences between the groups. The depth of anesthesia is known to influence intubating conditions: therefore, all variables of anesthesia were standardised. The size of the groups was similar to those in other studies dealing with this topic [10, 11, 15], and the rate of excellent intubating conditions (73%) was comparable to the results of other authors observing 70 to 80% [16, 17]. However, we were not able to find differences between both measurement sites with regard to intubating conditions. A reason for this result might be that most patients had maximum neuromuscular blocks in spite of low dose of rocuronium in both groups. Thus, the high percentage of acceptable conditions is not surprising.

Another limitation might be the restriction on female patients. Women usually require lower doses of neuromuscular blocking agents than men. Therefore, our approach made the groups more homogenous. On the other hand, the inclusion of male patients might have increased the rate of inadequate intubating conditions because of an insufficient neuromuscular block.

## Conclusions

Performing acceleromyography at the trapezius muscle reduced the time interval between induction of anesthesia and tracheal intubation by 18 s (11%). Thus, trapezius muscle acceleromyography is an acceptable alternative to adductor pollicis muscle acceleromyography in predicting acceptable intubating conditions, which allows for earlier indication of adequate intubating conditions.

## Abbreviations

ASA: American Society of Anesthesiologists physical status
BMI: Body Mass Index
Group A: neuromuscular measurements performed at the adductor pollicis muscle
Group T: neuromuscular measurements performed at the trapezius muscle
T1: The first of four consecutive twitches during assessment of the train of four ratio
T4: The fourth of four consecutive twitches during assessment of the train of four ratio
TOF ratio: Train of four ratio
TOF: Train of four stimulation
